# Lobomycosis of the Lower Limb in an Amazonian Patient

**DOI:** 10.4269/ajtmh.14-0748

**Published:** 2015-10-07

**Authors:** Mardelson Nery de Souza, Andreus Roberto Schlosser, Mônica da Silva-Nunes

**Affiliations:** Centro de Ciências da Saúde e do Desporto, Universidade Federal do Acre, Rio Branco, Acre, Brazil

**Figure 1.**
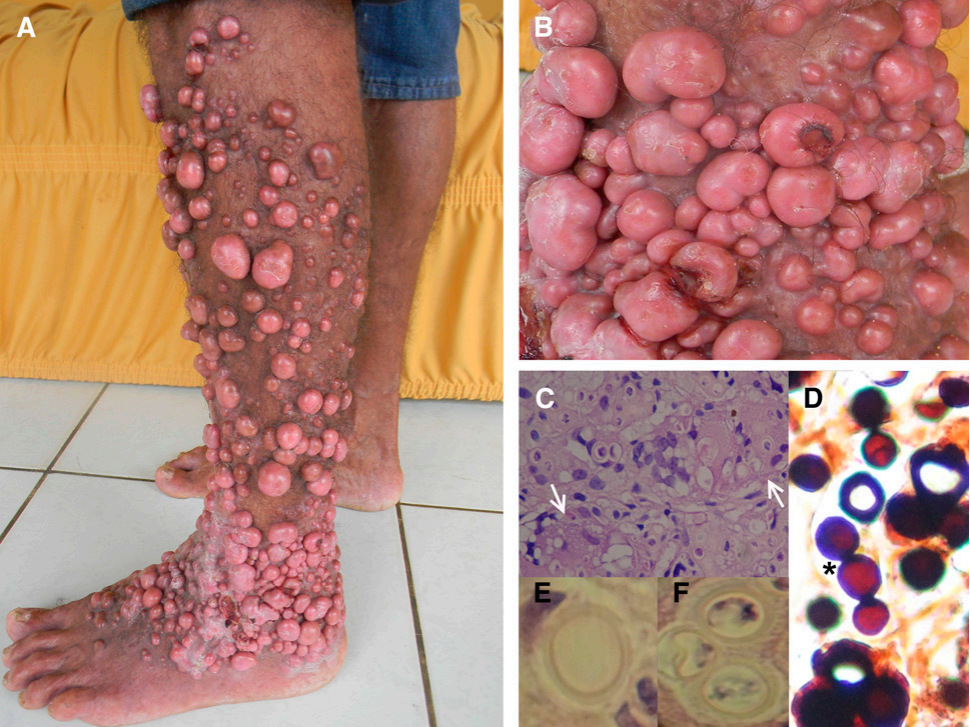
(**A**) Hyperplastic lesions in the lower left limb; (**B**) hyperplastic ulcerated lesions; (**C**) biopsy showing chronic granulomatous inflammation with forms of *Lacazia loboi* inside giant cells; (**D**) Grocott stain showing round yeasts with a double wall, forming single chains; (**E**) and (**F**) hematoxylin and eosin (HE) stain showing round yeasts with double wall at higher magnification.

A 56 year-old human immunodeficiency virus (HIV)–negative Amazonian male farmer presented with multiple skin nodules in the left lower limb, which started 28 years ago after a penetrating trauma in the lower limb with the thorn of a plant (Figure 1A

). He complained of frequent itching, ulceration, and bleeding of the nodules (Figure 1B). A biopsy revealed a chronic granulomatous process (Figure 1C, arrows) affecting the dermis, with rounded fungi with a thick double wall, forming single chains (Figure 1C–F, asterisk), and fulfilling the morphologic diagnostic criteria of *Lacazia loboi*.^1^ Lobomycosis is endemic in the Amazon region and is suspected when long-lasting dermal, keloid-like lesions are present in patients from the rainforest or farmers, and diagnosis is based on the biopsy findings. Differential diagnosis includes lepromatous and reactional tuberculoid leprosy, verrucous cutaneous leishmaniasis, chromomycosis, sporotrichosis, and keloids. The patient was referred for surgical resection of the nodules at the Dermatology State Service.

Treatment of lobomycosis is very difficult. Until recently, no efficient drug treatment was available for this disease. Recently, Bustamante and others^2^ described successful treatment with posaconazole in a patient that received 400 mg 2 × a day for 27 months. Woods and others^3^ also described successful drug treatment of lobomycosis in 10 patients that had leprosy. As the patients received regular treatment of leprosy with rifampicin, clofazimine, and dapsone, lobomycosis lesions decreased in size, and remaining lesions were excised.

Since lobomycosis is only seen in certain geographical regions of the world, and usually in poorly developed areas, there is a substantial lack of scientific knowledge, and more research is needed on treatment of this disease.
